# Load-dependent increase in lumbar kyphosis is associated with posterior pelvic tilt during deadlift

**DOI:** 10.3389/fspor.2025.1682991

**Published:** 2025-10-21

**Authors:** Kazuma Shoji, Koichi Nakayama, Masayo Shiouchi, Yoshiaki Manabe

**Affiliations:** ^1^ Graduate School of Health and Sport Sciences, Chukyo University, Aichi, Japan; ^2^ School of Health and Sport Sciences, Chukyo University, Aichi, Japan

**Keywords:** biomechanics, motion analyses, resistance training, deadlift exercise, spine biomechanics, lumbar lordosis angle, lumbar kyphosis angle, pelvic tilt angle

## Abstract

**Introduction:**

Although numerous previous studies have examined how different loading weights affect lumbar intervertebral discs during deadlift, the specific characteristics of trunk movement during these lifts remain unclear. This study aimed to compare how varying load weights affect trunk motion during deadlift, utilizing a model that accounts for the trunk's multi-degree-of-freedom motion.

**Methods:**

Thirteen participants performed standard deadlift at 60%, 70%, 80%, and 90% of their one repetition maximum (1RM). Reflective markers were placed on specific anatomical landmarks, including the tips of six spinous processes, and measured using an optical motion capture system. We then constructed a six-region link segment model of the trunk to calculate kinematic data for each spinal region in the sagittal plane. These data were subsequently compared across the different load weights.

**Results:**

The lower thoracic and upper lumbar regions showed increased flexion angle displacements as load weight increased. Additionally, the pelvis's posterior tilt accelerated with heavier loads.

**Discussion:**

While flexing the lumbar spine during lifting can be an effective strategy for successfully completing high-load deadlift, it may increase stress on the lumbar intervertebral discs. Therefore, maintaining lumbar spine lordosis and anterior pelvic tilt while ensuring trunk rigidity is important during high-load deadlift.

## 1 Introduction

Deadlift are widely used resistance training exercises that recruit multiple muscle groups, particularly the hip and trunk extensors, and impose substantial mechanical stress on the spine (1–4). This mechanical demand increases with load intensity (5–7), requiring precise trunk control to stabilize the body and transfer kinetic energy from the lower to the upper body (3, 8, 9). Heavier loads increased postural instability in resistance exercises such as the back squat, placing greater demands on trunk control mechanisms (10). Understanding trunk kinematics under varying load intensities is essential, given the trunk's critical role in deadlift performance.

Previous studies have examined the mechanical demands of deadlift using biomechanical analyses, primarily focusing on trunk kinetics (6, 7, 11, 12). Swinton et al. assessed L5/S1 net moments across relative loads (10% 1RM to 80% 1RM; 1RM: one repetition maximum) between straight bar and hexagonal bar deadlift. With the straight bar, peak lumbar net moment increased from 245 ± 46.3 Nm at 10% 1RM to 446.9 ± 73.9 Nm at 80% 1RM (7). Yanagisawa et al. reported that the apparent diffusion coefficient (ADC) at L5/S1 decreased between pre- and post-test under high-load conditions. Moreover, the ADC decrease at L5/S1 was significantly greater than at L1/2, L2/3, and L3/4 (12). These results suggest that mechanical stress on the lower spine increases with load intensity. Kinetics change based on the relative position of each vertebral body (13–15); therefore, examining the kinematics of the lumbar region, where excessive mechanical stress is applied, is considered a critical task directly linked to safe and effective training instructions.

Although deadlift kinematics have been widely studied, most research has focused on trunk inclination (7, 16, 17) or on comparing the lengths of the trunk segments across different exercises (18). However, only a few studies have specifically investigated the lumbar region or used multi-segment trunk models to assess intersegmental motion (6, 19). This is likely due to the common use of simplified models that represent the trunk as a single rigid segment, despite the spine's inherently complex, multi-joint structure (20). To enable more accurate analysis of spinal motion, advanced biomechanical models incorporating multiple trunk segments have been proposed and validated (21–24). For example, Kudo et al. (22) reported that increasing the number of segments improves the accuracy of detecting angular displacement during trunk movement. Accordingly, representing the trunk with only a few linked rigid segments may underestimate its actual deformation during dynamic tasks. Our previous work using a model that incorporates spinal mobility demonstrated that conventional deadlift elicits significantly greater lumbar flexion than parallel squats under high-load conditions (24). These findings suggest that multi-segment trunk models provide a more precise understanding of load-dependent spinal motion.

Segmental analysis of the trunk has practical relevance in both athletic and clinical settings. The thoracolumbar region is subject to increased mechanical stress during high-load lifting (5, 12, 18, 24). However, the changes in the thoracolumbar and lumbopelvic regions throughout the process leading up to high-load conditions remain unclear. Clarifying how each spinal segment responds to changing loads could inform injury prevention strategies, technique refinement, and personalized training prescriptions. With the growing prevalence of resistance training among both athletes and the general population, the demand for precise, segment-level biomechanical insights has become increasingly evident.

This study examined the effect of lifting load on trunk motion during deadlift, using a linked-segment model that divided the trunk into multiple segments. We hypothesized that with increasing load, flexion would increase in the lower spine with a greater range of motion and that this change would be associated with posterior pelvic tilt.

## 2 Materials and methods

### 2.1 Research design

This study investigated the effects of load intensity on segmental trunk motion during the deadlift. While prior research has often modeled the trunk as a single rigid segment, this simplification may obscure segment-specific compensatory strategies, particularly in regions exposed to high mechanical stress (7, 16–18). Thoracolumbar rounding and lumbar flexion occur under maximal load conditions, and the lumbar spine, with its high mobility, may be particularly sensitive to increased load demands. Therefore, a multi-segment model was adopted to provide a detailed biomechanical analysis of spinal kinematics.

A within-subjects design was used, in which each participant performed deadlift at four load intensities: 60%, 70%, 80%, and 90% of their 1RM. At each load, three repetitions were performed, and the second repetition was selected for analysis to avoid potential variability in the first lift and fatigue effects in the third. Motion capture data were collected using a 12-camera three-dimensional motion capture system to calculate angular displacement in six spinal regions during the lifting phase. The independent variable was load intensity (% 1RM), and the dependent variable was angular displacement in the six defined spinal regions. This approach allowed for precise within-participant comparisons of how increasing the mechanical load affected segmental trunk motion.

### 2.2 Participants

Thirteen male university track and field athletes (age: 20.6 ± 1.5 years; height: 175.2 ± 4.2 cm; body mass: 69.9 ± 4.9 kg; deadlift 1RM: 138.1 ± 22.6 kg) participated in this study. All participants had at least two training sessions per week and prior experience with deadlift, ensuring minimal need for exercise instruction. Inclusion criteria required participants to be free from musculoskeletal injuries at the time of testing and to have had no unresolved injuries within the previous 3 months.

The participants were informed about the purpose, benefits, and potential risks of the study. Written informed consent was obtained following oral explanation. The study protocol was approved by the *** University Research Ethics Committee for Studies Involving Human Samples (Approval No. 2021-041).

### 2.3 Procedures

All participants performed a 10 min dynamic warm-up before the 1RM deadlift test. The exercises were not standardized; instead, participants selected their own routines (e.g., dynamic stretching, mobility drills, jogging, body weight training). This procedure was adopted to reflect their habitual preparation and to prevent potential discomfort from an unfamiliar warm-up protocol. Subsequently, the 1RM was directly measured using an incremental loading protocol. This was followed by attempts at progressively heavier loads ranging from 5.0 to 0.5 kg until they achieved their actual 1RM (25). The experimental task consisted of a conventional deadlift (3, 26). Participants adopted a stance with hip-width foot placement (approximately 20–30 cm between heels). To ensure consistency of lifting form, an experienced investigator supervised each trial. Lifts showing evident form deviations were excluded from the analysis, and all lifts were performed using standardized equipment and setup.

The load intensities were set at 60%, 70%, 80%, and 90% of the 1RM. These load intensities follow the recommendations of Schoenfeld et al. (27). In their systematic review and meta-analysis, they reported that moderate loads (60%–80% 1RM) are most effective for hypertrophy, while heavier loads (80%–100% 1RM) are optimal for strength gains. Therefore, the selected range covers the intensities commonly used in resistance training programs aimed at improving both muscle hypertrophy and strength. Rest intervals of approximately 3‒5 min were provided between sets. At each load intensity, participants performed three repetitions. The second repetition was analyzed. This decision was made to avoid variability in the first trial due to insufficient familiarization and to minimize fatigue effects in the third trial, especially at heavier loads (e.g., 90% 1RM).

Participants were instructed to refrain from strenuous physical activity for 24 h prior to testing. No specific controls were imposed on hydration, nutrition, or sleep status; however, all tests were conducted within a consistent time window (±1 h) for each individual to minimize the influence of circadian variation.

### 2.4 Data collection and processing

**Figure 1:**
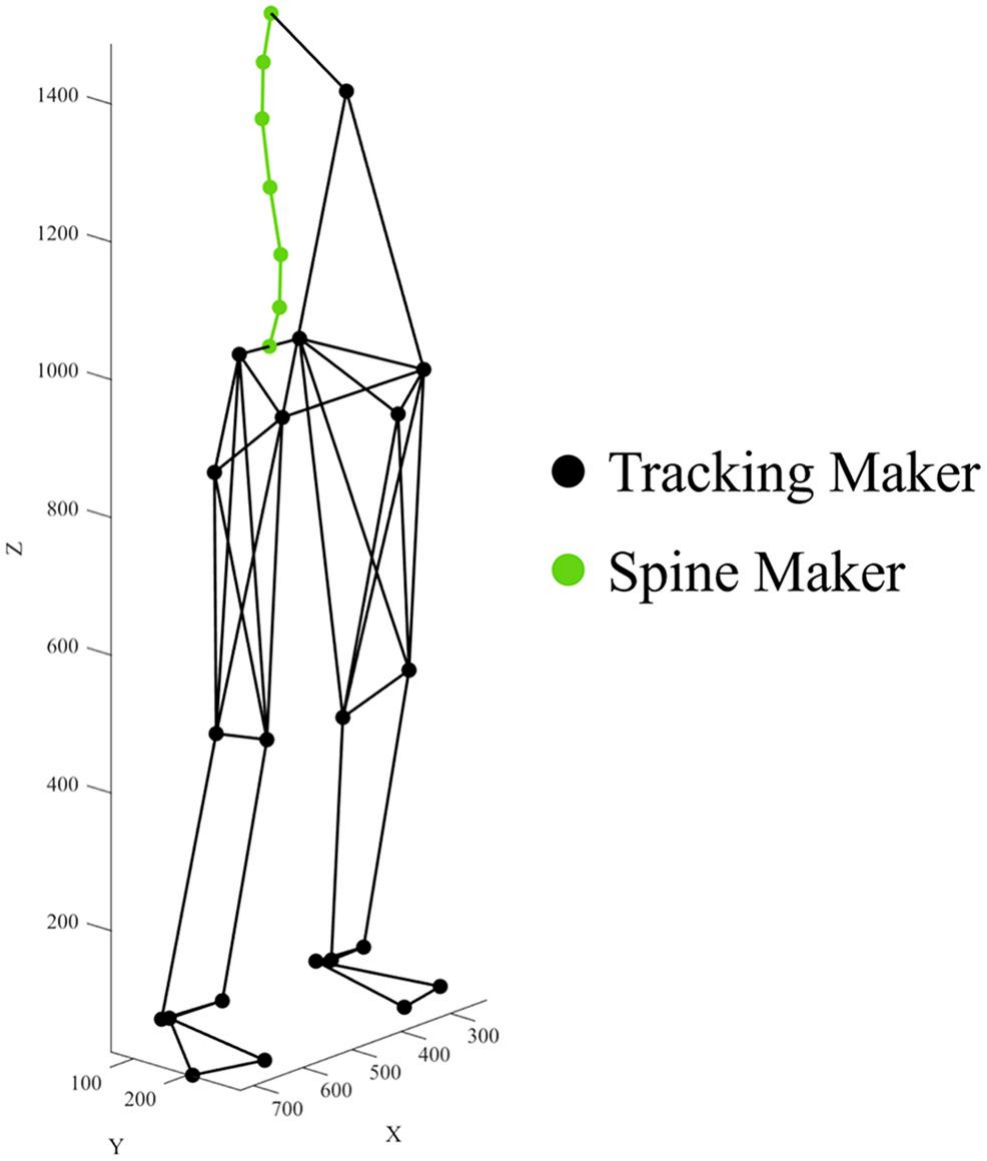
Maker placements.

Movement data were collected using a 12-camera three-dimensional motion capture system (Vicon MX; Vicon Motion Systems, Oxford, UK) at 250 Hz. Retroreflective markers were attached to 27 anatomical landmarks, including the spinous processes of the vertebrae, pelvis, and lower limbs (Figure 1). Marker locations were determined by palpation by an experienced examiner trained in spinal anatomy. The palpator had undergone palpation training and was highly skilled in spinal palpation. Furthermore, the palpator placed markers on all participants. Marker trajectories were filtered using a Butterworth low-pass digital filter with cutoff frequencies ranging from 13.7 to 38.8 Hz (28). The filtered 3D data were projected onto the sagittal plane to derive the 2D coordinates.

**Figure 2:**
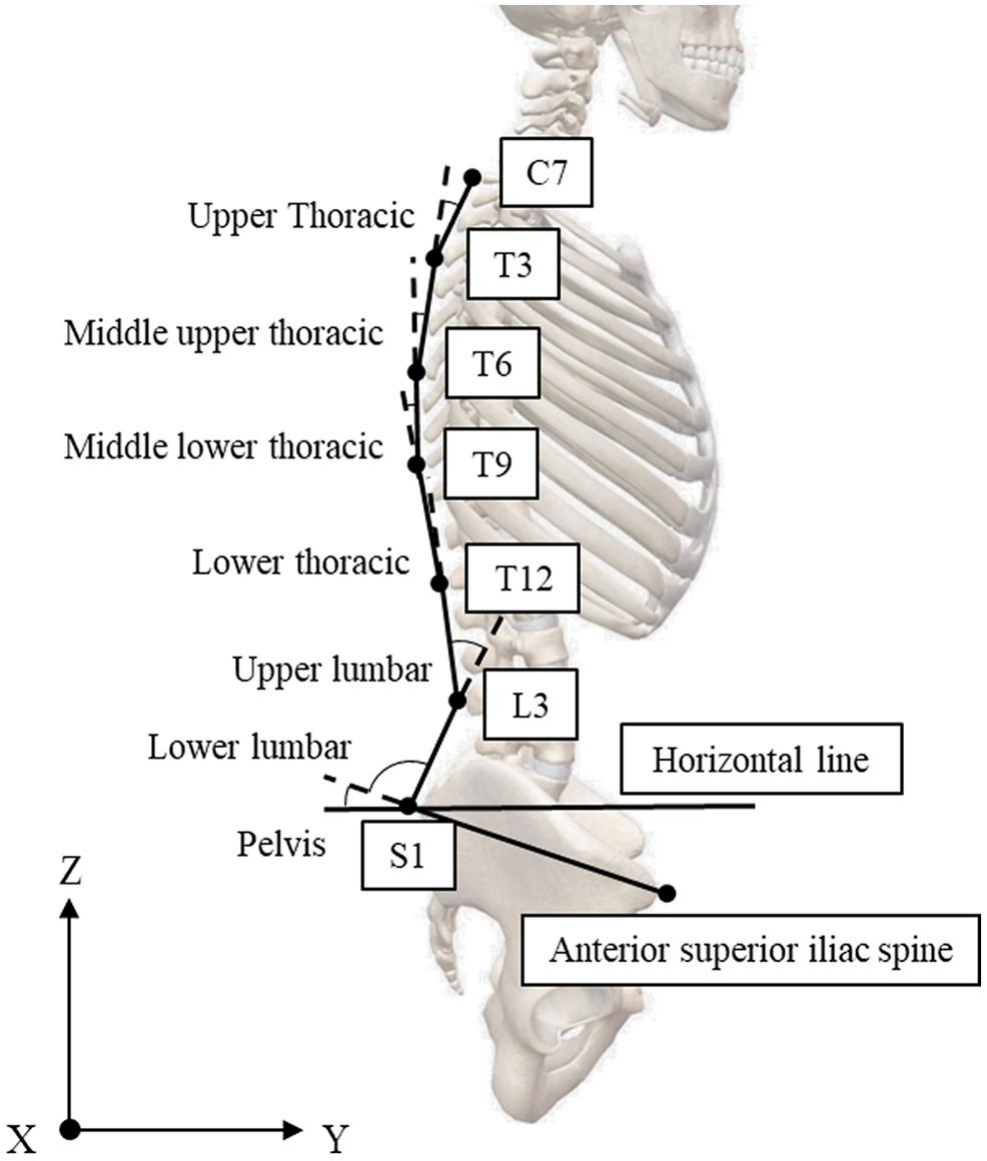
Link segment model of the trunk divided into six spinal regions.

Trunk motion was modeled using six segments based on spinous processes (Figure 2), following Shoji et al. (24). The segments are defined as follows:

▪ Seventh cervical vertebra (C7)–Third thoracic vertebra (T3)
▪ T3–Sixth thoracic vertebra (T6)
▪ T6–Ninth thoracic vertebra (T9)
▪ T9–Twelfth thoracic vertebra (T12)
▪ T12–Third lumbar vertebra (L3)
▪ L3–First sacral vertebra (S1) *S1–Posterior superior iliac spine (PSIS) midpoint
▪ S1–Anterior superior iliac spine (ASIS) midpoint

Angular displacement was calculated for six spinal regions using the intersegmental angle formed by two adjacent trunk segments. Each segmental angle was defined as the internal angle between two vectors connecting consecutive anatomical landmarks (e.g., the upper thoracic angle was defined as the angle between the C7–T3 and T3–T6 segments). The reference posture for angle calculation was a neutral standing posture (Table 1). Segmental angles were expressed as changes from the reference position. Extension was represented by positive values, and flexion by negative values. The pelvic angle was defined as the angle between the horizontal line through the PSIS and the line segment connecting the PSIS and ASIS. All angles were computed in the sagittal plane using 2D projected coordinates. Time normalization was applied to the lifting phase: 0% at the lowest center of mass (COM) of the trunk, 50% when the bar passed the knees, and 100% at the highest COM of the trunk. The COM of the trunk segment was calculated using the body part inertia coefficient of Japanese athletes (29). In addition, the moment arm between the hip joint center and the barbell center was defined as the horizontal distance, calculated as the difference in their *Y*-axis positions.

**Table 1:** Reference spinal angles in the standing posture.

Spine region	Mean ± SD	95% CI
Upper thoracic angle	167.61 ± 3.87	165.17–170.04
Middle upper thoracic angle	170.55 ± 3.14	168.58–172.53
Middle lower thoracic angle	168.79 ± 4.20	166.15–171.44
Lower thoracic angle	188.26 ± 5.84	184.60–191.94
Upper lumbar angle	198.41 ± 4.73	195.43–201.39
Lower lumbar angle	86.48 ± 4.15	83.88–89.09
Pelvic tilt angle	10.93 ± 3.13	8.96–12.90

Values are given as mean (SDs) in degree.

### 2.5 Statistical analysis

Statistical analyses were performed using SPSS (version 29.0, SPSS Inc., Chicago, IL, USA). For discrete data, all datasets satisfied the assumption of normality; therefore, a one-way repeated measures ANOVA was used to compare differences in the hip-to-barbell moment arm across load conditions. *post hoc* comparisons between conditions were conducted using Bonferroni-adjusted *p*-values. The level of significance in SPSS analyses was set at *p* < 0.05.

For continuous data, Statistical Parametric Mapping (SPM) implemented in MATLAB (Wellcome Trust Center for Neuroimaging, London, UK) was used. The normality of each dataset in SPM analysis was also assessed using the Shapiro–Wilk test, with parametric or non-parametric tests applied as appropriate. A one-way repeated measures ANOVA was conducted to examine differences in angular displacement across load conditions. When significant effects were observed, Bonferroni-corrected paired *t*-tests were performed in accordance with Pataky et al. (30). The significance level was set at *p* = 0.05 for ANOVA and *p* = 0.0083 for *post hoc* comparisons.

The partial eta squared (*η*_p_^2^) was calculated for the ANOVA and interpreted as: trivial (<0.010), small (0.010‒0.059), moderate (0.060‒0.140), and large (>0.140). Cohen's *d* was interpreted as: small (0.20‒0.49), medium (0.50‒0.79), large (>0.80).

## 3 Results

**Figure 3:**
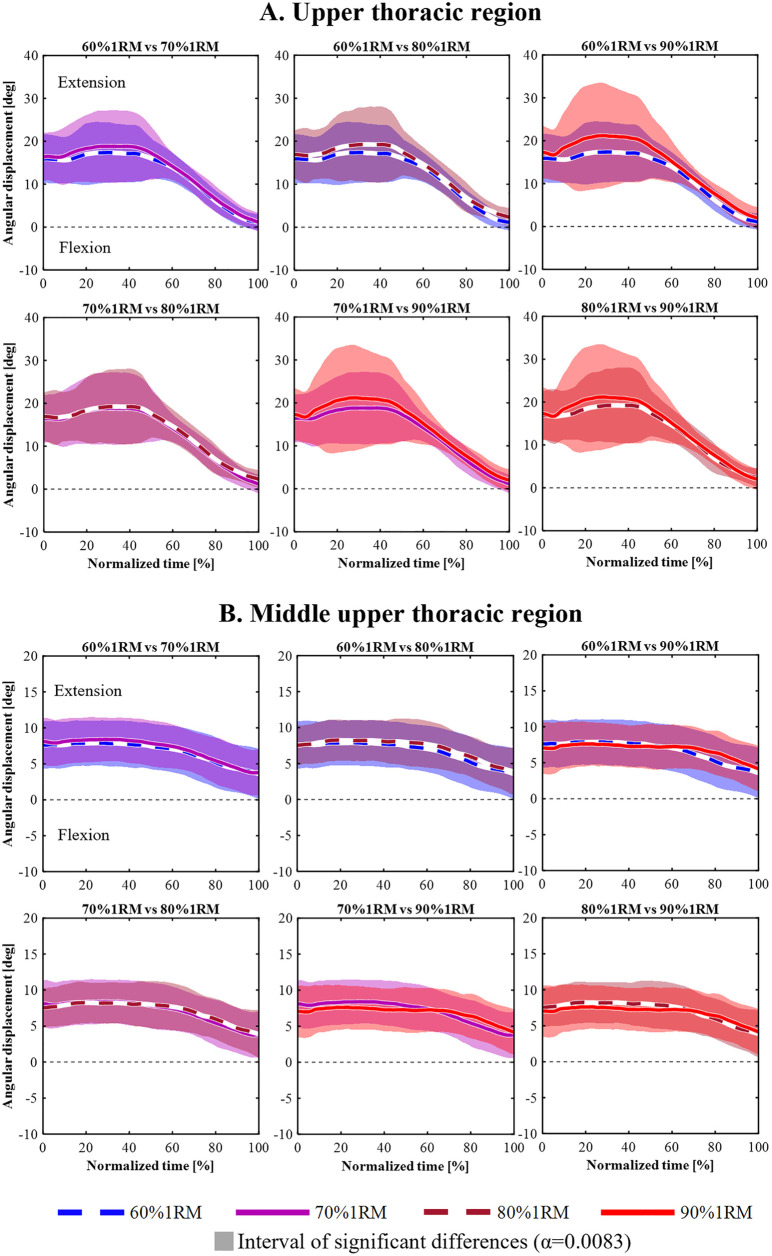
Time-series angular displacement data of upper thoracic **(A)** and middle upper thoracic regions **(B)** across four load intensities during the lifting phase.

**Figure 4:**
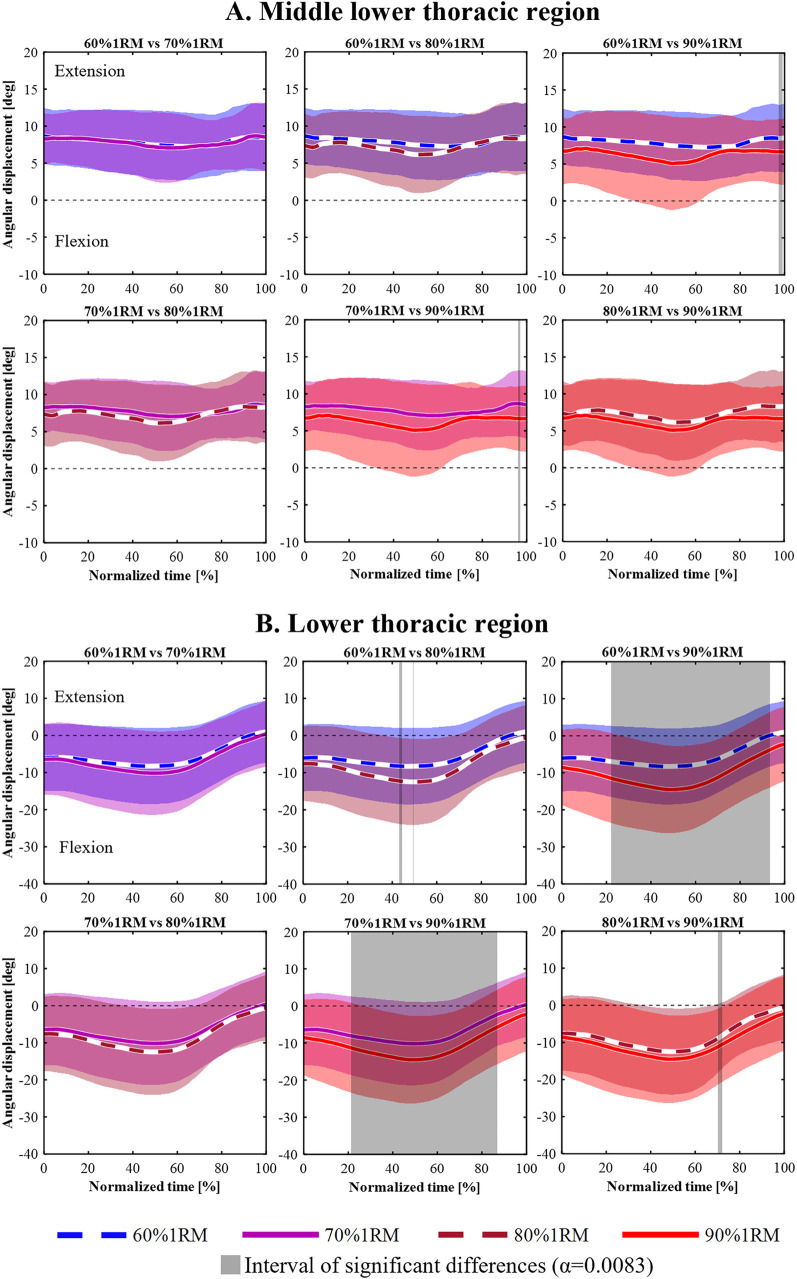
Time-series angular displacement data of middle lower thoracic **(A)** and lower thoracic regions **(B)** across four load intensities during the lifting phase.

**Figure 5:**
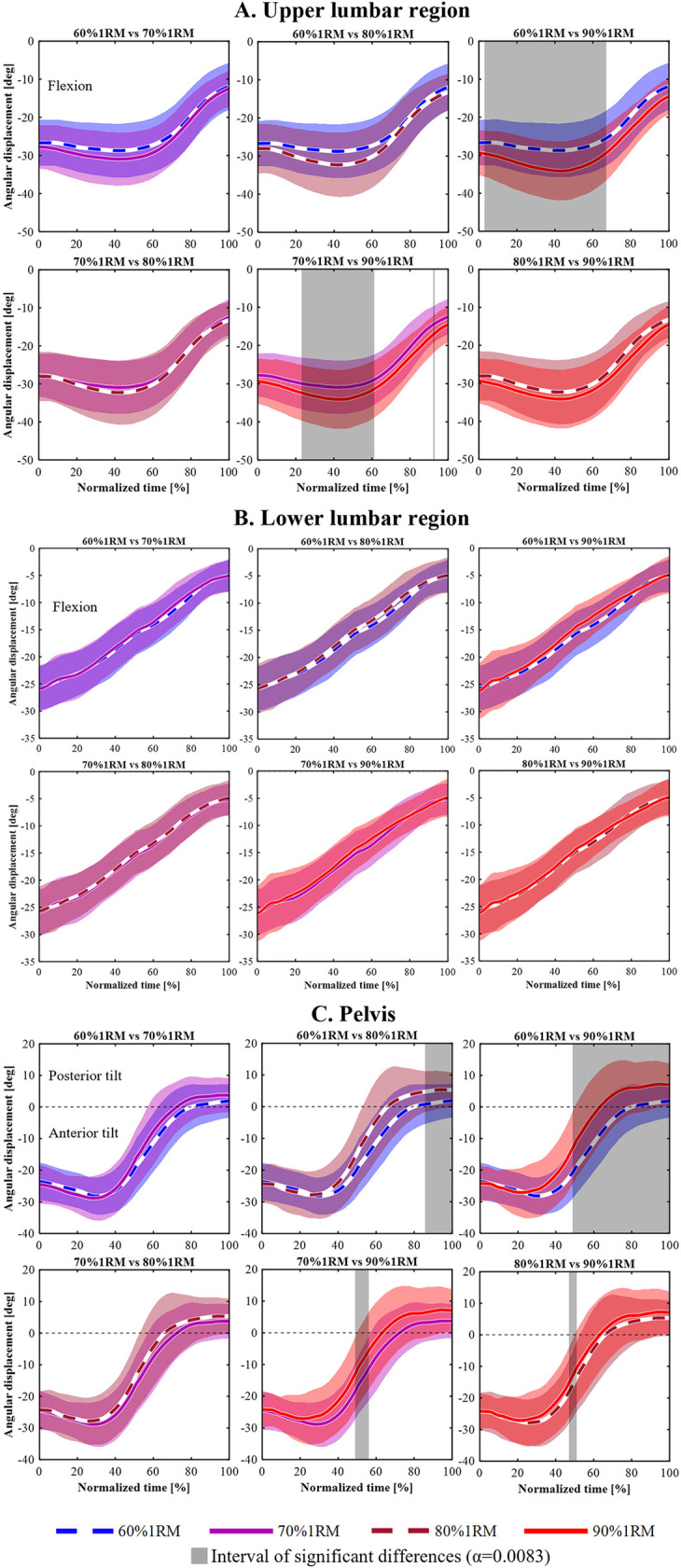
Time-series angular displacement data of upper lumbar **(A)**, lower lumbar **(B)**, and pelvic regions **(C)** across four load intensities during the lifting phase.

Figures 3–5 shows the mean angular displacement for each spinal region across different load intensities, calculated as the difference between dynamic and standing reference postures. One-way repeated measures ANOVA using SPM revealed significant effects of load intensity on the angular displacement of the upper thoracic, middle lower thoracic, lower thoracic, upper lumbar, lower lumbar, and pelvis (*p* < 0.05). In contrast, no significant differences were observed in the middle upper thoracic regions (Figure 3B). *post hoc* comparisons using Bonferroni-corrected paired t-tests were conducted for regions showing significant main effects.

For the upper thoracic region (Figure 3A), a significant main effect was observed in 9%–12% and 32%–39% of the lifting phase (*p* = 0.020, *p* = 0.040, respectively). However, Bonferroni-corrected *post hoc* comparisons revealed no significant differences among the four load conditions.

For the middle lower thoracic (Figure 4A), a significant main effect was observed in 35%–58% and 89%–100% of the lifting phase (*p* = 0.030, *p* = 0.044, respectively). However, Bonferroni-corrected *post hoc* comparisons revealed no significant differences among the four load conditions.

For the lower thoracic (Figure 4B), a significant main effect of load intensity was observed in 0%–100% of the lifting phase (*p* < 0.001). *post hoc* analyses showed that the 80% 1RM condition produced significantly greater flexion angles than the 60% 1RM condition during 43%–44% and 48% of the lifting phase (*p* = 0.008, *p* = 0.008, respectively). The 90% 1RM condition showed significantly greater flexion angles than the 60% 1RM condition during 22%–94% of the lifting phase (*p* < 0.001). The 90% 1RM condition showed significantly greater flexion angles than the 70% 1RM condition during 21%–87% of the lifting phase (*p* < 0.001). Furthermore, during the 70%–72% lifting phase, the 90% 1RM condition showed significantly greater flexion than the 80% 1RM condition (*p* = 0.007).

For the upper lumbar (Figure 5A), a significant main effect of load intensity was observed in 0%–74% and 78%–100% of the lifting phase (*p* = 0.002, *p* = 0.037, respectively). *post hoc* analyses showed that the 90% 1RM condition produced significantly greater flexion angles than the 60% 1RM condition during 3%–67% of the lifting phase (*p* < 0.001). Additionally, the 90% 1RM condition showed significantly greater flexion compared to the 70% 1RM condition during 23%–61% and 92%–93% of the lifting phase (*p* < 0.001, *p* = 0.008, respectively).

For the lower lumbar (Figure 5B), a significant main effect of load intensity was observed during 44%–46% and 55%–65% of the lifting phase (*p* = 0.049, *p* = 0.040, respectively). However, Bonferroni-corrected *post hoc* comparisons revealed no significant differences among the four load conditions.

For the pelvis (Figure 5C), a significant main effect of load intensity was observed during the 37%–100% lifting phase (*p* < 0.001). *post hoc* comparisons showed that the 80% 1RM condition produced significantly greater posterior tilt angle than the 60% 1RM condition during the 86%–100% lifting phase (*p* = 0.001). The 90% 1RM condition resulted in a significantly greater posterior tilt angle than the 60% 1RM condition during 49%–100% (*p* < 0.00). At 49%–56%, the 70% 1RM condition showed significantly greater anterior tilt angle than the 90% 1RM condition (*p* = 0.002), and at 47%–51%, the 80% 1RM condition showed significantly greater anterior tilt angle than the 90% 1RM condition (*p* = 0.004).

Table 2 shows the mean ± SD hip to barbell moment arm across different load conditions. A one-way repeated-measures ANOVA revealed significant differences across loads during 0%–100% of the lifting phase (*p* = 0.003, *η*_p_² = 0.419). *post hoc* comparisons indicated that the mean moment arm was shorter in the 90% 1RM condition than in the 60% 1RM (*p* = 0.031, *d* = 0.97) and 70% 1RM conditions (*p* = 0.014, *d* = 0.66), and shorter in the 80% 1RM condition than in the 60% 1RM condition (*p* = 0.050, *d* = 0.61). During 0%–20% of the lifting phase, significant differences were also found across loads (*p* = 0.029, partial *η*² = 0.243), with *post hoc* tests showing a shorter mean moment arm in the 70% 1RM condition than in the 60% 1RM condition (*p* = 0.031, *d* = 0.84). Furthermore, during 61%–80% of the lifting phase, the ANOVA revealed significant differences across loads (*p* = 0.001, partial *η*² = 0.465). *post hoc* comparisons showed that the mean moment arm in the 90% 1RM condition was significantly shorter than in the 60% 1RM (*p* = 0.037, *d* = 0.98), 70% 1RM (*p* = 0.002, *d* = 0.93), and 80% 1RM conditions (*p* = 0.031, *d* = 0.64).

**Table 2:** Hip-barbell moment arm (cm): comparison across different load conditions.

Load (%1RM)	Normalized time (%)																				
	0%–20%				21%–40%			41%–60%			61%–80%				81%–100%			0%–100%			
	Mean	±	SD		Mean	±	SD	Mean	±	SD	Mean	±	SD		Mean	±	SD	Mean	±	SD	
60% 1RM	29.73	±	1.27	*	29.61	±	1.34	27.45	±	1.75	21.56	±	1.78	^‡^	14.43	±	2.28	24.59	±	1.10	^†^ ^,^ ^‡^
70% 1RM	28.85	±	0.80		28.72	±	1.19	27.07	±	1.28	21.53	±	1.99	^‡^	14.59	±	2.16	24.20	±	1.10	^‡^
80% 1RM	28.86	±	0.98		29.03	±	0.64	26.95	±	1.28	20.88	±	2.07	^‡^	13.90	±	1.55	23.97	±	0.91	
90% 1RM	28.64	±	1.14		28.70	±	1.35	26.36	±	1.88	19.35	±	2.64		13.52	±	1.82	23.37	±	1.40	

Bonferroni-adjusted *p*-values (<0.05 considered significant).

* vs. 70% 1RM.

^†^ vs. 80% 1RM.

^‡^ vs. 90% 1RM.

## 4 Discussion

This study examined the effect of lifting load trunk motion during deadlift, using a linked-segment model that divided the trunk into multiple segments. We hypothesized that with increasing load, flexion would increase in the lower spine with a greater range of motion and that this change would be associated with posterior pelvic tilt. SPM analysis revealed that, with increasing load, flexion angles increased in the lower thoracic and upper lumbar spine. Additionally, the transition to posterior pelvic tilt occurred earlier under heavier load conditions. These findings support the hypotheses proposed in this study.

The deadlift involves lifting a barbell from the floor along the legs with straight arms until the knees, hips, and shoulders are fully extended. To reduce the risk of lumbar injury, maintaining slight lumbar lordosis and anterior pelvic tilt is commonly recommended throughout the movement in clinical and coaching settings (4, 31).

However, our findings showed that under high-load conditions, participants deviated from this recommended posture when performing the lift (Figures 4B, 5A). These findings are consistent with those reported by Hales et al. and Shoji et al., who observed increased spinal flexion during high-load deadlift conditions (18, 24). Proud et al. observed significantly greater flexion in the middle-lower thoracic, lower thoracic, and upper lumbar segments as load increased (23). Boocock et al. further demonstrated that with repeated lifting, lumbosacral and trunk flexion increased significantly, from 71.7% to 98.4% and 63.9% to 87.7%, respectively (32). These findings suggest that increased lifting load and muscular fatigue contribute to lumbar flexion, potentially compromising the ability to maintain recommended alignment.

The greater range of motion in the lumbar spine compared to the thoracic spine may be attributed to differences in anatomical structure. White and Panjabi reported that the lumbar spine has greater flexion-extension mobility due to structural distinctions from the thoracic spine (20). Thoracic motion is restricted by anatomical features such as the rib cage and long spinous processes, which inherently limit segmental mobility in this region. In contrast, the lumbar spine has sagittally oriented facet joints and a relatively high disc-to-body height ratio, both of which enhance mobility (20, 33). Thus, when the trunk adapts to increasing load demands, the highly mobile lumbar spine tends to flex preferentially to support both stability and mechanical efficiency.

The observed increase in lumbar flexion with higher loads suggests that this adaptation may serve to shorten the moment arm and improve mechanical leverage, thereby enhancing lifting efficiency. In this study, the hip-to-barbell moment arm was significantly shorter under heavier loads during 61%–80% of the lifting phase, where the 90% 1RM condition showed the shortest values among the other loads. During deadlift, the barbell exerts a flexion moment on the trunk, requiring activation of trunk and hip extensors to maintain extension moment and rigidity (34). Under high-load conditions, the trunk flexion moment induced by the load weight may exceed the muscle-generated extension moment, potentially compromising the ability to maintain trunk rigidity. Thus, insufficient trunk extension torque under high-loads likely led to spinal flexion, particularly rounding of the lumbar region. This postural change shortened the distance between the barbell and hip joint center, thereby reducing the required trunk extension moment and enabling the lift. One possible contributor to lumbar rounding is early posterior pelvic tilt under high-load conditions. In this study, heavier loads induced earlier posterior tilt of the pelvis, leading to kyphotic curvature of the upper spine and increased lumbar flexion. These findings support the hypotheses proposed in this study.

In this context, Swinton et al. further suggested that sumo-style deadlift reduce hip torque demands by shortening the moment arm between the barbell and hip through a more upright posture (7). Hales et al. observed greater spinal rounding in deadlift, accompanied by shorter trunk segment lengths (0.49 ± 0.04 m) compared to squats (0.54 ± 0.07 m) (18). While these findings concern lifting style differences, they consistently indicate that reducing the moment arm contributes to improved efficiency and lower torque demands. Accordingly, the increased flexion observed in the lower thoracic and upper lumbar spine may reflect a mechanically adaptive strategy to reduce trunk flexion moment.

Currently, no consistent evidence exists regarding the relationship between lumbar flexion and lumbar injury during upright lifting. Rounding of the lumbar spine shortens the back extensor moment arm, reducing trunk extension torque (12). Consequently, mechanical load shifts from muscles to passive tissues like vertebrae and discs, increasing intradiscal pressure and the risk of lumbar injury (35). Moreover, increased trunk flexion from a neutral posture contributes to elevated shear forces (36), as observed in this study. Von Arx et al. reported that squat lifting produces higher shear forces at the L5/S1 level than stoop lifting with a bent back and therefore considered squat lifting dangerous (37). However, in that study, only the starting position of the weight was fixed, and the trajectory of the weight was not analyzed. The squat lift may have lifted the weight straight upward from the upright starting position, whereas the stoop lift may have lifted the weight closer to the body. Although evidence linking lumbar flexion to lumbar injury remains inconsistent, lumbar flexion is generally considered to increase mechanical stress on the lumbar intervertebral discs.

Conversely, lumbar flexion may help increase intra-abdominal pressure (IAP), thereby contributing to trunk stability. To maintain a neutral spine under high-load, co-contraction of trunk muscles and IAP elevation through the Valsalva maneuver are required (38–41). However, under high-load conditions, maintaining a neutral spine becomes difficult, potentially leading to compensatory trunk flexion. Trunk flexion displaces abdominal contents upward, pushing the diaphragm superiorly and increasing pressure at the thoracoabdominal boundary. This may passively raise IAP, thereby supplementing extensor torque and enhancing trunk stiffness (42). Use of a lifting belt also increases IAP and spinal stiffness through abdominal compression (43, 44). Thus, increased lumbar flexion under high-loads may reflect a compensatory lifting strategy that promotes stability via IAP regulation. In this study, although lumbar rounding occurred with increased lifting load, it was a result of the lifting strategy and not something that can be linked to lumbar injury.

## 5 Limitations

This study has certain limitations. First, participants were limited to young, trained males. Therefore, caution should be exercised when generalizing the findings, as factors such as sex, age, and training history may influence the results. Second, the use of skin-mounted markers and palpation-based identification of anatomical landmarks may result in measurement errors due to soft tissue artifacts (STA). Johnson et al. demonstrated inaccuracies caused by STA through combined CT scanning and motion capture, suggesting the need for such techniques to more precisely capture skeletal motion (45). However, in the current study, marker placement was fixed, and all lifting conditions were analyzed using the same procedures. Therefore, although STA was consistently present, it is unlikely to have significantly affected the results when comparing relative differences between conditions. Third, although muscle activity and IAP are potential explanatory factors for movement adaptations, this study focused on time series data of spinal kinematics under different load conditions. We therefore restricted our analyses to angular displacements. The inclusion of kinetic outcomes (e.g., joint torques, powers) and direct neuromuscular or physiological measures (e.g., surface electromyography, pressure sensors) would provide further insights. Future research should integrate these measures to clarify the mechanisms underlying such kinematic adaptations.

## 6 Conclusion

Increased load intensity in deadlift induces segment-specific movement changes, particularly in the lower thoracic spine, upper lumbar spine, and pelvis. Under high-load conditions, increased lumbar flexion and posterior pelvic tilt appear to be closely linked and may reflect complex interactions among lifting efficiency, anatomical characteristics, and neuromuscular adaptation. Understanding the conditions under which these adaptations emerge is essential for optimizing lifting technique and minimizing injury risk in strength training.

## 7 Practical applications

These findings provide practical implications for both athletic and clinical settings. Coaches and practitioners should recognize that high-load deadlift induce greater lumbar flexion and earlier posterior pelvic tilt. When these changes become excessive, they may increase spinal loading and the risk of injury. At the same time, such postural adjustments can shorten the hip-to-barbell moment arm and improve lifting efficiency. Therefore, training instructions should emphasize maintaining lumbar lordosis and anterior pelvic tilt during the early and middle phases of the lift to prevent injury while enhancing strength. In competitive situations where lifting maximal loads is required, adopting lumbar flexion and posterior pelvic tilt can reduce the moment arm and provide a mechanical advantage. However, the potential injury risk in this case must be carefully considered.

## Data Availability

The original contributions presented in the study are included in the article/Supplementary Material, further inquiries can be directed to the corresponding author.
